# 2-[(2-Carboxy­phen­yl)sulfan­yl]acetic acid

**DOI:** 10.1107/S1600536809034126

**Published:** 2009-09-05

**Authors:** Cai-Hong Zhan, Ling-Xian Chen, Yun-Long Feng

**Affiliations:** a Zhejiang Key Laboratory for Reactive Chemistry on Solid Surfaces, Institute of Physical Chemistry, Zhejiang Normal University, Jinhua, Zhejiang 321004, People’s Republic of China

## Abstract

The title compound, C_9_H_8_O_4_S, affords a zigzig chain in the crystal structure by inter­molecular O—H⋯O hydrogen bonds. The molecular geometry suggests that extensive but not uniform π-electron delocalization is present in the benzene ring and extends over the exocyclic C—S and C—C bonds.

## Related literature

For background to the coordination chemistry of rigid carboxyl­ate system, see: Sagatys *et al.* (2003 ▶); Sokolov *et al.* (2001 ▶).
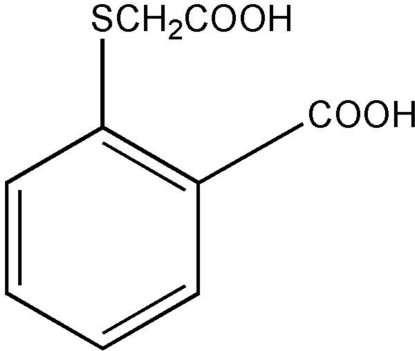


## Experimental

### 

#### Crystal data

C_9_H_8_O_4_S
*M* *_r_* = 212.22
Triclinic, 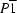

*a* = 5.1786 (5) Å
*b* = 9.2973 (9) Å
*c* = 10.4776 (11) Å
α = 69.980 (4)°
β = 81.959 (6)°
γ = 79.732 (6)°
*V* = 464.69 (8) Å^3^
*Z* = 2
Mo *K*α radiation
μ = 0.33 mm^−1^
*T* = 296 K
0.33 × 0.24 × 0.15 mm

#### Data collection

Bruker APEXII diffractometer
Absorption correction: multi-scan (*SADABS*; Sheldrick, 1996 ▶) *T*_min_ = 0.910, *T*_max_ = 0.952
6609 measured reflections
2110 independent reflections
1941 reflections with *I* > 2σ(*I*)
*R*_int_ = 0.024

#### Refinement

*R*[*F* ^2^ > 2σ(*F* ^2^)] = 0.074
*wR*(*F* ^2^) = 0.222
*S* = 1.19
2110 reflections
133 parameters
2 restraints
H atoms treated by a mixture of independent and constrained refinement
Δρ_max_ = 0.65 e Å^−3^
Δρ_min_ = −0.34 e Å^−3^

### 

Data collection: *APEX2* (Bruker, 2002 ▶); cell refinement: *SAINT* (Bruker, 2002 ▶); data reduction: *SAINT*; program(s) used to solve structure: *SHELXS97* (Sheldrick, 2008 ▶); program(s) used to refine structure: *SHELXL97* (Sheldrick, 2008 ▶); molecular graphics: *SHELXTL* (Sheldrick, 2008 ▶); software used to prepare material for publication: *SHELXTL*.

## Supplementary Material

Crystal structure: contains datablocks I, global. DOI: 10.1107/S1600536809034126/at2858sup1.cif

Structure factors: contains datablocks I. DOI: 10.1107/S1600536809034126/at2858Isup2.hkl

Additional supplementary materials:  crystallographic information; 3D view; checkCIF report

## Figures and Tables

**Table 1:** Hydrogen-bond geometry (Å, °)

*D*—H⋯*A*	*D*—H	H⋯*A*	*D*⋯*A*	*D*—H⋯*A*
O2—H2⋯O1^i^	0.86 (7)	1.92 (5)	2.687 (6)	149 (8)
O3—H3⋯O4^ii^	0.85 (2)	1.80 (5)	2.634 (4)	167 (6)

Symmetry codes: (i) 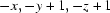; (ii) 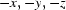.

**Fig. 1.**
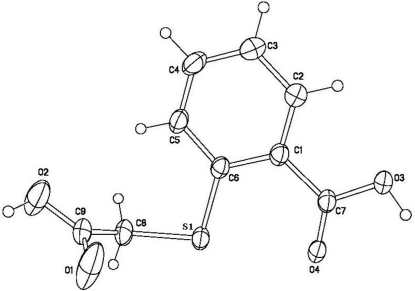
The molecular structure of the title compound, showing 30% probability displacement ellipsoids

## supplementary crystallographic information

### Comment

Thioacetatebenzoic acid (I) is an interesting ligand from a structural point of view since it can display a wide range of coordination patterns with metal ions. The ligand (I) belongs to dicarboxylic acids. The characteristic coordination chemistry of the rigid carboxylate system may facilitate the formation of inorganic-organic materials with high thermal stability and form large channels, while the peculiar coordination chemistry of the flexible carboxylate system employed in the self-assembly reaction has versatile coordination behavior and may be favorable for the formation of the helical structure (Sagatys *et al.*, 2003; Sokolov *et al.*, 2001). As shown in Fig.1, the bond lengths within the benzene ring exhibit the expected pattern with C—C bonds (1.368 (8)–1.399 (6) Å) between the single and double bonds. And the bond distance of C1—C7 (1.476 (5) Å) and S1—C6 (1.768 (4) Å) also fall between the double and single bonds. All these interatomic distances suggest that extensive but not uniform π electron delocalization is present in the benzene ring and extends over the exocyclic C—S and C—C bonds. The torsion angle of C6—S1—C8—C9 is -71.7 (4)°. O—H···O hydrogen bonds link independent molecules to form a zigzig chain.

### Experimental

To an aqueous solution of 2-thiobenzoic acid (1.54 g, 10.0 mmol) and NaOH (0.80 g, 20.0 mmol) were sequentially added the aqueous solution of chloroactic acid (2.835 g, 30.0 mmol) and NaOH (1.400 g, 35.0 mmol). After stirring for 4 h at 353 K under nitrogen atmosphere, the mixture was cooled to room temperature slowly. Adjusted the pH to 2 by adding 1.0 mol/*L* HCl, the pink deposit appeared rapidly. The solids were filtered and washed with water. The single crystals suitable for X-ray diffraction were obtained by the recrystallization of sieved solid in the ethanol.

### Refinement

The H atoms bonded to C atoms were positioned geometrically [aromatic C—H = 0.93 Å and aliphatic C—H = 0.97 Å, *U*_iso_(H) = 1.2*U*_eq_(C)]. The H atoms bonded to O atoms were located in a difference Fourier map and refined freely.

### Crystal data

**Table:**

C_9_H_8_O_4_S	*Z* = 2
*M**_r_* = 212.22	*F*(000) = 220
Triclinic, *P*1	*D*_x_ = 1.517 Mg m^−^^3^
Hall symbol: -P 1	Mo *K*α radiation, λ = 0.71073 Å
*a* = 5.1786 (5) Å	Cell parameters from 5343 reflections
*b* = 9.2973 (9) Å	θ = 2.1–27.7°
*c* = 10.4776 (11) Å	µ = 0.33 mm^−^^1^
α = 69.980 (4)°	*T* = 296 K
β = 81.959 (6)°	Block, colourless
γ = 79.732 (6)°	0.33 × 0.24 × 0.15 mm
*V* = 464.69 (8) Å^3^	

### Data collection

**Table:**

Bruker APEXII diffractometer	2110 independent reflections
Radiation source: fine-focus sealed tube	1941 reflections with *I* > 2σ(*I*)
graphite	*R*_int_ = 0.024
ω scans	θ_max_ = 27.7°, θ_min_ = 2.1°
Absorption correction: multi-scan (*SADABS*; Sheldrick, 1996)	*h* = −6→6
*T*_min_ = 0.910, *T*_max_ = 0.952	*k* = −12→11
6609 measured reflections	*l* = −13→12

### Refinement

**Table:**

Refinement on *F*^2^	Primary atom site location: structure-invariant direct methods
Least-squares matrix: full	Secondary atom site location: difference Fourier map
*R*[*F*^2^ > 2σ(*F*^2^)] = 0.074	Hydrogen site location: inferred from neighbouring sites
*wR*(*F*^2^) = 0.222	H atoms treated by a mixture of independent and constrained refinement
*S* = 1.19	*w* = 1/[σ^2^(*F*_o_^2^) + (0.0736*P*)^2^ + 1.2589*P*] where *P* = (*F*_o_^2^ + 2*F*_c_^2^)/3
2110 reflections	(Δ/σ)_max_ < 0.001
133 parameters	Δρ_max_ = 0.65 e Å^−^^3^
2 restraints	Δρ_min_ = −0.34 e Å^−^^3^

### Special details

**Table:**

Geometry. All e.s.d.'s (except the e.s.d. in the dihedral angle between two l.s. planes) are estimated using the full covariance matrix. The cell e.s.d.'s are taken into account individually in the estimation of e.s.d.'s in distances, angles and torsion angles; correlations between e.s.d.'s in cell parameters are only used when they are defined by crystal symmetry. An approximate (isotropic) treatment of cell e.s.d.'s is used for estimating e.s.d.'s involving l.s. planes.
Refinement. Refinement of *F*^2^ against ALL reflections. The weighted *R*-factor *wR* and goodness of fit *S* are based on *F*^2^, conventional *R*-factors *R* are based on *F*, with *F* set to zero for negative *F*^2^. The threshold expression of *F*^2^ > σ(*F*^2^) is used only for calculating *R*-factors(gt) *etc*. and is not relevant to the choice of reflections for refinement. *R*-factors based on *F*^2^ are statistically about twice as large as those based on *F*, and *R*- factors based on ALL data will be even larger.

### Fractional atomic coordinates and isotropic or equivalent isotropic displacement parameters (Å^2^)

**Table:**

	*x*	*y*	*z*	*U*_iso_*/*U*_eq_	
S1	0.2028 (2)	0.36823 (12)	0.11601 (11)	0.0417 (3)	
O1	−0.0678 (11)	0.4660 (9)	0.3612 (6)	0.116 (2)	
O2	0.2828 (9)	0.5474 (7)	0.3870 (5)	0.0891 (16)	
H2	0.173 (12)	0.569 (10)	0.449 (6)	0.107*	
O3	0.2508 (7)	−0.1074 (4)	0.1122 (4)	0.0549 (9)	
H3	0.127 (8)	−0.107 (7)	0.067 (5)	0.066*	
O4	0.0891 (7)	0.1410 (4)	0.0369 (3)	0.0506 (8)	
C1	0.4065 (8)	0.0579 (5)	0.2004 (4)	0.0383 (9)	
C2	0.5712 (9)	−0.0691 (6)	0.2747 (5)	0.0482 (10)	
H2A	0.5667	−0.1659	0.2682	0.058*	
C3	0.7421 (10)	−0.0540 (7)	0.3583 (5)	0.0571 (12)	
H3A	0.8511	−0.1398	0.4081	0.068*	
C4	0.7485 (10)	0.0890 (7)	0.3665 (5)	0.0571 (13)	
H4A	0.8638	0.1000	0.4221	0.068*	
C5	0.5879 (9)	0.2166 (6)	0.2942 (5)	0.0465 (10)	
H5A	0.5959	0.3124	0.3018	0.056*	
C6	0.4120 (8)	0.2051 (5)	0.2093 (4)	0.0365 (8)	
C7	0.2342 (8)	0.0364 (5)	0.1093 (4)	0.0402 (9)	
C8	0.2613 (11)	0.5208 (5)	0.1742 (5)	0.0488 (11)	
H8A	0.4498	0.5209	0.1687	0.059*	
H8B	0.1864	0.6192	0.1130	0.059*	
C9	0.1482 (10)	0.5076 (6)	0.3179 (5)	0.0504 (11)	

### Atomic displacement parameters (Å^2^)

**Table:**

	*U*^11^	*U*^22^	*U*^33^	*U*^12^	*U*^13^	*U*^23^
S1	0.0549 (7)	0.0366 (5)	0.0400 (6)	−0.0082 (4)	−0.0092 (4)	−0.0178 (4)
O1	0.093 (3)	0.210 (7)	0.109 (4)	−0.084 (4)	0.041 (3)	−0.121 (5)
O2	0.076 (3)	0.149 (5)	0.077 (3)	−0.034 (3)	0.002 (2)	−0.075 (3)
O3	0.064 (2)	0.0412 (17)	0.073 (2)	0.0014 (15)	−0.0322 (18)	−0.0301 (16)
O4	0.063 (2)	0.0398 (16)	0.060 (2)	−0.0022 (14)	−0.0285 (16)	−0.0241 (15)
C1	0.040 (2)	0.043 (2)	0.038 (2)	−0.0067 (17)	−0.0030 (16)	−0.0202 (17)
C2	0.052 (3)	0.046 (2)	0.050 (3)	−0.004 (2)	−0.012 (2)	−0.020 (2)
C3	0.056 (3)	0.062 (3)	0.055 (3)	0.000 (2)	−0.021 (2)	−0.020 (2)
C4	0.046 (3)	0.077 (3)	0.060 (3)	−0.005 (2)	−0.020 (2)	−0.033 (3)
C5	0.042 (2)	0.058 (3)	0.054 (3)	−0.012 (2)	−0.0053 (19)	−0.033 (2)
C6	0.0368 (19)	0.044 (2)	0.0344 (19)	−0.0082 (16)	−0.0008 (15)	−0.0194 (16)
C7	0.043 (2)	0.041 (2)	0.045 (2)	−0.0066 (17)	−0.0047 (17)	−0.0241 (18)
C8	0.067 (3)	0.038 (2)	0.050 (2)	−0.016 (2)	−0.008 (2)	−0.0189 (19)
C9	0.058 (3)	0.047 (2)	0.060 (3)	−0.010 (2)	−0.009 (2)	−0.032 (2)

### Geometric parameters (Å, °)

**Table:**

S1—C6	1.768 (4)	C2—C3	1.385 (6)
S1—C8	1.809 (4)	C2—H2A	0.9300
O1—C9	1.224 (7)	C3—C4	1.368 (8)
O2—C9	1.251 (6)	C3—H3A	0.9300
O2—H2	0.86 (7)	C4—C5	1.372 (7)
O3—C7	1.315 (5)	C4—H4A	0.9300
O3—H3	0.85 (2)	C5—C6	1.399 (6)
O4—C7	1.216 (5)	C5—H5A	0.9300
C1—C2	1.388 (6)	C8—C9	1.509 (7)
C1—C6	1.409 (6)	C8—H8A	0.9700
C1—C7	1.476 (5)	C8—H8B	0.9700
			
C6—S1—C8	103.4 (2)	C6—C5—H5A	119.4
C9—O2—H2	105 (6)	C5—C6—C1	117.6 (4)
C7—O3—H3	105 (4)	C5—C6—S1	121.8 (3)
C2—C1—C6	120.0 (4)	C1—C6—S1	120.6 (3)
C2—C1—C7	118.8 (4)	O4—C7—O3	122.1 (4)
C6—C1—C7	121.2 (4)	O4—C7—C1	123.9 (4)
C3—C2—C1	121.0 (4)	O3—C7—C1	114.1 (4)
C3—C2—H2A	119.5	C9—C8—S1	114.5 (3)
C1—C2—H2A	119.5	C9—C8—H8A	108.6
C4—C3—C2	119.1 (5)	S1—C8—H8A	108.6
C4—C3—H3A	120.5	C9—C8—H8B	108.6
C2—C3—H3A	120.5	S1—C8—H8B	108.6
C3—C4—C5	121.1 (4)	H8A—C8—H8B	107.6
C3—C4—H4A	119.4	O1—C9—O2	122.5 (5)
C5—C4—H4A	119.4	O1—C9—C8	121.3 (4)
C4—C5—C6	121.2 (4)	O2—C9—C8	116.1 (5)
C4—C5—H5A	119.4		

### Hydrogen-bond geometry (Å, °)

**Table:**

*D*—H···*A*	*D*—H	H···*A*	*D*···*A*	*D*—H···*A*
O2—H2···O1^i^	0.86 (7)	1.92 (5)	2.687 (6)	149 (8)
O3—H3···O4^ii^	0.85 (2)	1.80 (5)	2.634 (4)	167 (6)

Symmetry codes: (i) −*x*, −*y*+1, −*z*+1; (ii) −*x*, −*y*, −*z*.
